# Mitochondrial-Targeted Two-Photon Fluorescent Probes for Zinc Ions, H_2_O_2_, and Thiols in Living Tissues

**DOI:** 10.1155/2013/323619

**Published:** 2013-01-31

**Authors:** Hwan Myung Kim, Bong Rae Cho

**Affiliations:** ^1^Division of Energy Systems Research, Ajou University, Suwon 443-749, Republic of Korea; ^2^Department of Chemistry, Korea University, 1-Anamdong, Seoul 136-701, Republic of Korea

## Abstract

Mitochondria provide the energy of the cells and are the primary site of oxygen consumption and the major source of reactive oxygen species. In mitochondria, metal ions and glutathione play vital roles in maintaining their structure and the redox environment. To understand their roles in mitochondria, it is crucial to monitor each of these chemical species in the mitochondria at the cell, tissue, and organism levels. An ideal tool for such purpose is the use of two-photon microscopy (TPM). Until recently, however, there has been no report on the two-photon (TP) probes suitable for such applications. In this paper, we summarize the mitochondria-targeted TP probes for Zn^2+^, H_2_O_2_, and thiols, as well as their bioimaging applications.

## 1. Introduction 

**Scheme 1:**
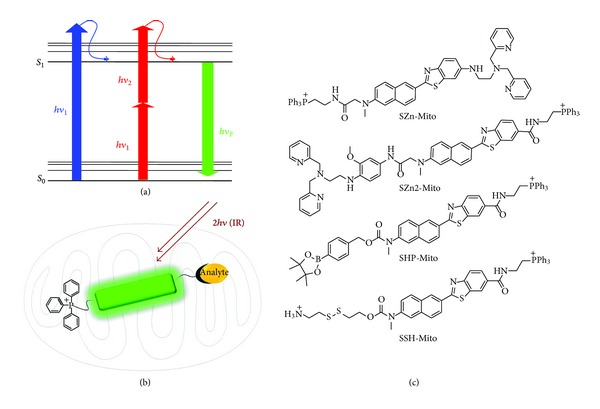
(a) Comparison of one- and two-photon exited fluorescence; the fluorescence is emitted upon excitation by one-photon of higher energy and two-photons of lower energy, respectively. (b) Design of the mitochondria-targeted two-photon probes. Mitochondrial targeting moiety (TPP) and receptors for the analytes are linked to the opposite ends of the fluorophore. (c) Chemical structures of the mitochondrial-targeted two-photon probes for Zn^2+^ (SZn-Mito and SZn2-Mito), H_2_O_2_ (SHP-Mito), and thiols (SSH-Mito).

Mitochondria provide the energy of the cells. They are primary cellular compartments of oxygen consumption and the major source of reactive oxygen species (ROS) [1, 2]. In mitochondria, metal ions and glutathione (GSH) play vital roles in maintaining their structure and the redox environment [3–6]. To understand the physiology of mitochondria, it is crucial to monitor such chemical species in mitochondria at the cell, tissue, and organism levels. For this purpose, a number of one-photon fluorescent probes, derived from fluorescein or rhodamine as the fluorophore and various receptors, have been developed [7–9]. However, most of these probes have been evaluated with one-photon microscopy (OPM), which uses single photon of higher energy as the excitation source (Scheme 1(a)). This requirement limited their application in live tissue imaging owing to the shallow penetration depth (less than 80 *μ*m), photobleaching, and cellular autofluorescence.

An attractive approach to the detection of biologically important species deep inside live tissues is the use of two-photon microscopy (TPM). TPM, a new technique which employs two near-infrared photons as the excitation source (Scheme 1(a)), has become an indispensable tool in biology and medicine due to the advantages it offers. They include deeper penetration depth (>500 *μ*m), lower tissue auto-fluorescence and self-absorption, reduced photodamage and photobleaching, in addition to the intrinsically localized excitation [13–16]. This allows molecular imaging deep inside the intact tissue for a long period of time with minimum interference from the tissue preparation artifacts which can extend >70 *μ*m into the tissue interior [17]. However, the progress in this field is limited by the lack of two-photon (TP) probes. As such, many biologists are using one photon fluorescent probes for TPM imaging, despite that most of them have too low two-photon (TP) cross-sections (*δ*_TPA_ < 50 GM) to be useful for TPM [18]. Therefore, there is a pressing need to develop a variety of TP probes for specific applications [15, 16].

To meet such demands, we have developed a series of mitochondrial-targeted TP probes that can selectively detect mitochondrial Zn^2+^ (SZn-Mito, SZn2-Mito), H_2_O_2_ (SHP-Mito), and thiols (SSH-Mito) in live tissues (Scheme 1(c)) [10–12, 19]. These probes are derived from 6-(benzo [*d*]thiazol-2′-yl)-2-(*N,N*-dimethylamino)naphthalene (BTDAN) as the TP fluorophore, triphenylphosphonium salt (TPP) as the mitochondrial targeting site [20, 21], and receptors for the specific analytes (Scheme 1(b)). TPP and the receptors have been separated as far as possible to minimize the interactions between them. In this paper, we summarize the photophysical properties and biological imaging applications of mitochondrial-targeted TP probes for Zn^2+^, H_2_O_2_, and thiols.

## 2. Two-Photon Probes for Mitochondrial Zinc Ion

 Zinc ion is the second most abundant d-block metal ion in the human brain and is an active component in enzymes and proteins [22–26]. For proper brain functions, it is vital to maintain Zn^2+^-ion homeostasis, which is controlled by the import of intracellular free Zn^2+^ ions ([Zn^2+^]_*i*_) from and export to the extracellular cellular space, the endoplasmic reticulum, and mitochondria. While mitochondria can take up evoked [Zn^2+^]_*i*_ rise, a strong elevation of intramitochondrial Zn^2+^ ([Zn^2+^]_*m*_) can promote mitochondrial dysfunctions [27, 28]. Recently, we have developed TP probes for [Zn^2+^]_*m*_ (SZn-Mito and SZn2-Mito, Scheme 1(c)) derived from BTDAN as the reporter, *N,N*-di-(2-picolyl)ethylenediamine (DPEN) as the Zn^2+^ chelator, [29, 30] and TPP as the mitochondrial targeting group.

SZn-Mito and SZn2-Mito are TP fluorescent turn-on probes based on the photoinduced electron transfer (PeT) process [31]. Upon addition of Zn^2+^, the fluorescence intensity of SZn-Mito and SZn2-Mito increased gradually presumably because of the blocking of the PeT upon binding with Zn^2+^. The TP fluorescence enhancement factor (FEF = (*F* − *F*_min⁡_)/*F*_min⁡_) of SZn-Mito was 7 in MOPS buffer (30 mM, pH 7.2), while that of SZn2-Mito was 68 in HEPES buffer (50 mM, pH 7.4) (Table 1) [10]. Noteworthy was the large (10-fold) increase in the FEF by the slight modification of the chemical structure. The dissociation constants (*K*_*d*_^OP^ and *K*_*d*_^TP^) of SZn-Mito and SZn2-Mito for the complexation with Zn^2+^ were 3.1 and 1.4 nM, respectively, regardless of the excitation mode (Table 1); thus, these probes can detect Zn^2+^ in the nanomolar range. Both probes exhibited high selectivity for 1 *μ*M Zn^2+^ compared with 1 mM of Na^+^, K^+^, Ca^2+^, Mg^2+^, 1 *μ*M of Mn^2+^, Fe^2+^, Co^2+^, and Pb^2+^ and modest selectivity over 1 *μ*M Ni^2+^and Cd^2+^ [10, 19]. In the presence of 1 *μ*M of Cu^2+^ and Hg^2+^, the fluorescence was quenched due to the metal-to-ligand electron transfer upon excitation [32]. Moreover, they were pH insensitive in the biologically relevant pH range. Since Ni^2+^, Cd^2+^, Hg^2+^, and Cu^2+^ ions rarely exist in the cells [33], these probes can detect Zn^2+^ with minimum interference from other competing metal ions and pH. Moreover, the TP action cross-sections (Φ*δ*) of SZn-Mito and SZn2-Mito were 75 and 155 GM at 760 and 750 nm, respectively, in the presence of excess Zn^2+^ (Table 1). The combined results reveal that SZn2-Mito is a more sensitive TP probe which can detect mitochondrial Zn^2+^ with higher sensitivity and twice as bright TPM image than SZn-Mito. 

**Figure 1:**
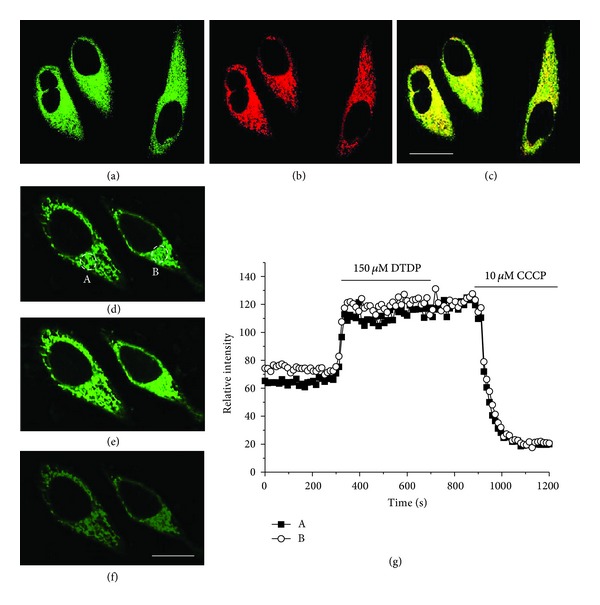
(a) TPM and (b) OPM images of HeLa cells colabeled with (a) SZn2-Mito (1 *μ*M) and (b) Mitotracker Red FM (1 *μ*M). (c) Colocalized image. The wavelengths for one- and two-photon excitations were 514 and 760 nm, respectively, and the emission was collected at 450–550 (SZn-Mito) and 600–700 nm (Mitotracker Red FM), respectively. Scale bar, 20 *μ*m. (d–f) TPM images of 1 *μ*M SZn2-Mito-labeled HeLa cells, before (d) and after (e) addition of 150 *μ*M DTDP to the imaging solution. (f) After addition of 10 *μ*M CCCP to (e). (g) The relative TPEF intensity of SZn2-Mito-labeled HeLa cells as a function of time. The TPEF intensities were collected at 450–600 nm upon excitation at 750 nm with fs pulse. Scale bar, 10 *μ*m. Cells shown are representative images from replicate experiments (*n* = 5) [10].

The utility of SZn2-Mito in the cell imaging was confirmed by the following experiments. First, the TPM and OPM images of HeLa cells colabeled with SZn2-Mito and Mitotracker Red FM, a well-known OP fluorescent probe for mitochondria [8], overlapped well. The Pearson's colocalization coefficient, *A*, calculated by using Autoquant X2 software, of SZn2-Mito with Mitotracker Red FM was 0.85 (Figures 1(a)–1(c)) [34]. Second, the TPEF intensity decreased dramatically when the probe-labeled cells were treated with *N,N,N′,N′*-tetrakis(2-pyridyl)ethylenediamine (TPEN), a membrane permeable Zn^2+^ chelator that can effectively remove [Zn^2+^]_*m*_ [35]. Third, SZn2-Mito showed negligible toxicity as measured by using CCK-8 kit and high photostability as indicated by the negligible change in the TP excited fluorescence (TPEF) intensity after continuous irradiation of the fs pulses for 60 min [10]. Fourth, the TPEF intensity increased dramatically when 2,2′-dithiodipyridine (DTDP; 150 *μ*M), a reagent that promotes the release of Zn^2+^ from Zn^2+^-binding proteins [36], was added to HeLa cells labeled with SZn2-Mito (Figures 1(d), 1(e), and 1(g)) and decreased abruptly upon addition of carbonyl cyanide *m*-chlorophenylhydrazone (CCCP; 10 *μ*M, Figures 1(f) and 1(g)), a compound that promotes the release of intramitochondrial cations by collapsing the mitochondrial membrane potential [37]. 

**Figure 2:**
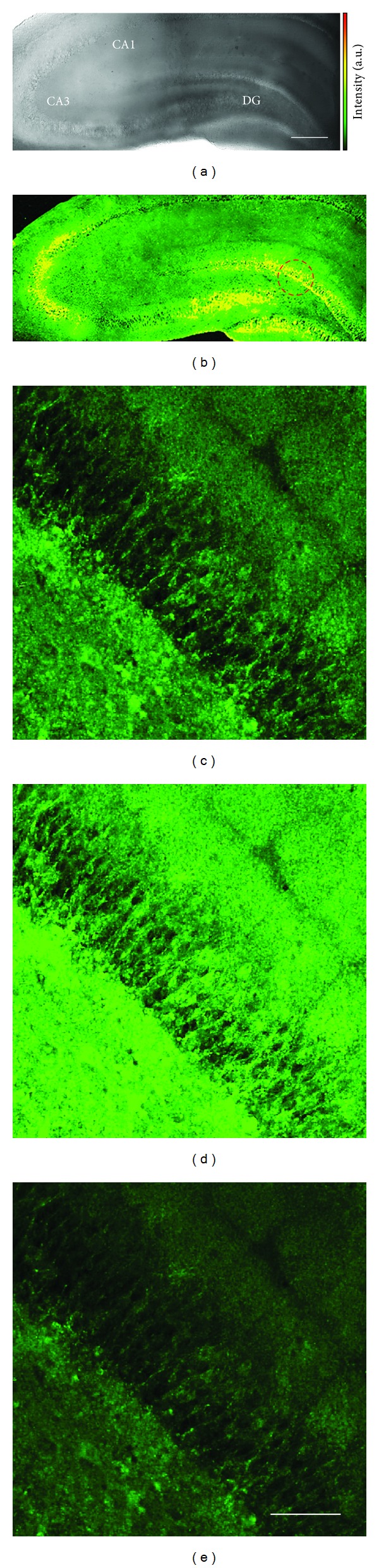
Images of a rat hippocampal slice stained with 20 *μ*M SZn2-Mito for 1 h. (a) Bright-field images of the CA1-CA3 regions as well as dentate gyrus (DG) at 10x magnification. (b) 10 TPM images along the *z*-direction at the depths of approximately 100–200 *μ*m were accumulated. (c–e) Magnification at 40x in the DG regions (red circle in (b)) at a depth of ~100 *μ*m (c) before and (d) after addition of 150 *μ*M DTDP to the imaging solution. (e) After addition of 10 *μ*M CCCP to (d). Scale bars: (a) 300 and (e) 75 *μ*m [10].

To assess the utility of SZn2-Mito in tissue imaging, fresh hippocampal slices from a 14-day-old rat were labeled with 20 *μ*M SZn2-Mito. The TPM image of the probe-labeled tissue revealed marked TPEF in the CA3 and DG regions at depths of 100–200 *μ*m (Figure 2(b)). At a higher magnification, the [Zn^2+^]_*m*_ distribution in the DG region was clearly visualized (Figure 2(c)). Moreover, the TPEF intensity increased after addition of DTDP and decreased upon treatment of CCCP, thereby confirming that the bright regions reflect the [Zn^2+^]_*m*_ (Figures 2(d) and 2(e)). These results established that SZn2-Mito is capable of detecting [Zn^2+^]_*m*_ at depths of 100–200 *μ*m in living tissues by using TPM [10]. 

## 3. Two-Photon Probe for Mitochondrial H_2_O_2_

**Scheme 2:**
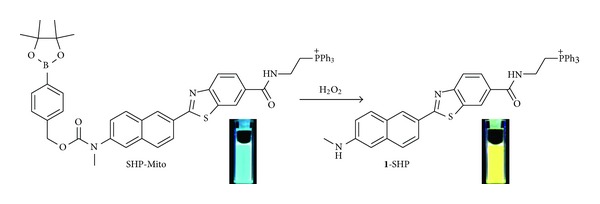
Structures of SHP-Mito and **1**-SHP [11].

Hydrogen peroxide (H_2_O_2_) is a prominent member of the ROS family and plays crucial roles in physiology, aging, and disease in living organisms [38–40]. While controlled bursts of H_2_O_2_ are utilized for cell signaling [41], abnormal production or accumulation of H_2_O_2_ within mitochondria has been linked to severe disorder such as cancer and neurodegenerative Alzheimer's and Parkinson's diseases [42–44]. To detect mitochondrial H_2_O_2_ deep inside live tissues, we have developed a ratiometric TP probe (SHP-Mito, Scheme 2) derived from BTDAN as the reporter, a boronate-based carbamate leaving group as the H_2_O_2_ response site, and TPP as the mitochondrial targeting site. We anticipated that the H_2_O_2_-triggered boronate cleavage of the electron poor carbamate linkage would liberate the more electron rich **1**-SHP, giving rise to red-shifted fluorescent emission (Scheme 2) [11].

 The emission spectra of a 1 *μ*M solution of SHP-Mito increased gradually at 530–600 nm (*F*_yellow_) with a concomitant decrease at 400–470 (*F*_blue_) nm in the presence of 1 mM H_2_O_2_ in MOPS buffer [11]. This process followed pseudo 1st-order kinetics with *k*_obs_ = 1.0–1.2 × 10^−3^ s^−1^ and resulted in a 75-fold enhancement in the *F*_yellow_/*F*_blue_ ratio. The detection limit of SPH-Mito for H_2_O_2_ was 4.6 *μ*M. Moreover, SHP-Mito exhibited high selectivity for H_2_O_2_ over competing ROS and reactive nitrogen species (RNS), as revealed by unperturbed *F*_yellow_/*F*_blue_ ratios upon addition of 200 *μ*M concentrations of various ROS and RNS, including *tert*-butyl hydroperoxide (TBHP), hypochlorite (OCl^−^), superoxide (O_2_^−^), nitric oxide (NO), *tert*-butoxy radical (^•^OBu^*t*^), hydroxyl radical (^•^OH), and peroxynitrite (ONOO^−^), and was pH insensitive at biologically relevant pH range [11]. The TP action (Φ*δ*) spectra of SHP-Mito and **1**-SHP in MOPS buffer (30 mM, pH 7.4) indicated Φ*δ*_max⁡_ values of 11 and 55 GM at 740 and 750 nm, respectively (Table 1). This predicts 5-fold brighter TPM images of the probe-labeled cells after complete conversion of SHP-Mito to **1**-SHP.

**Figure 3:**
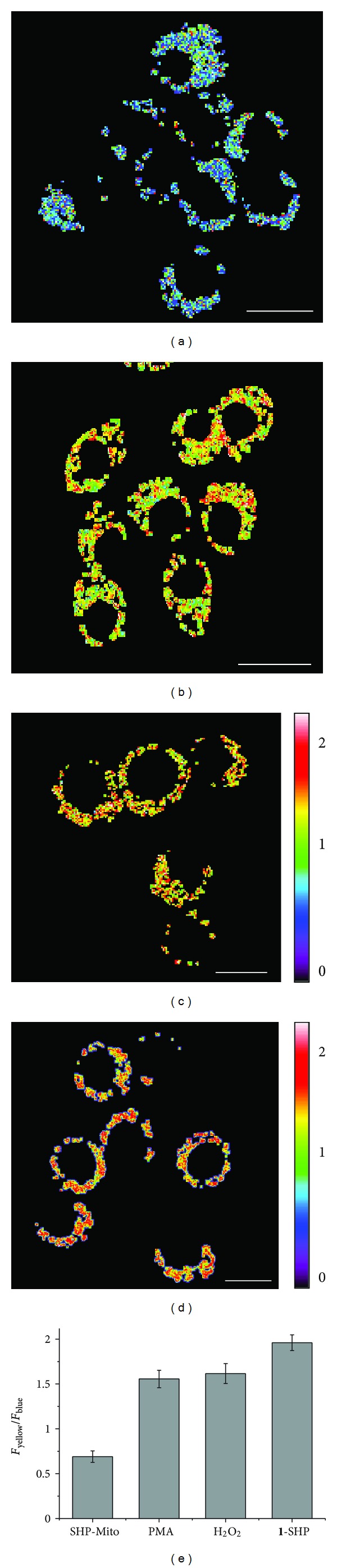
(a–d) Pseudocolored ratiometric TPM images (*F*_yellow_/*F*_blue_) of Raw 264.7 cells incubated with 3 *μ*M (a) SHP-Mito and (d) **1**-SHP. (b and c) Cells were pretreated with (b) PMA (1 *μ*g/mL) for 30 min and (c) 200 *μ*M H_2_O_2_ for 30 min. (e) Average *F*_yellow_/*F*_blue_ intensity ratios in figures (a–d). Images were acquired using 750 nm excitation and fluorescent emission windows: blue = 400–470 nm and yellow = 530–600 nm. Scale bar = 15 *μ*m. Cells shown are representative images from replicate experiments (*n* = 5) [11].

 The utility of SHP-Mito in the live cell imaging was established by the following experiments. First, the TPM and OPM images of Raw 264.7 murine macrophage cells co-labeled with SHP-Mito and MitoTracker Red merged well with the *A* of 0.91 [11]. Second, the ratiometric images constructed from two collection windows ranging from 400 to 470 nm (*F*_blue_) and 530 to 600 nm (*F*_yellow_) gave an average emission ratio of *F*_yellow_/*F*_blue_ = 0.62 and 1.96 for SHP-Mito and **1**-SHP, respectively (Figures 3(a), 3(d), and 3(e)). The ratio increased to 1.62 when the cells were preincubated for 30 min with 200 *μ*M H_2_O_2_ and to 1.56 upon treatment with phorbol myristate acetate (PMA), which induces H_2_O_2_ generation through a cellular inflammation response (Figures 3(b), 3(c), and 3(e)) [45]. These results confirmed that SHP-Mito is responsive to the change in the H_2_O_2_ concentration. Third, SHP-Mito shows nontoxicity to cells during the imaging experiments, as determined by using a CCK-8 kit [11]. 

**Figure 4:**
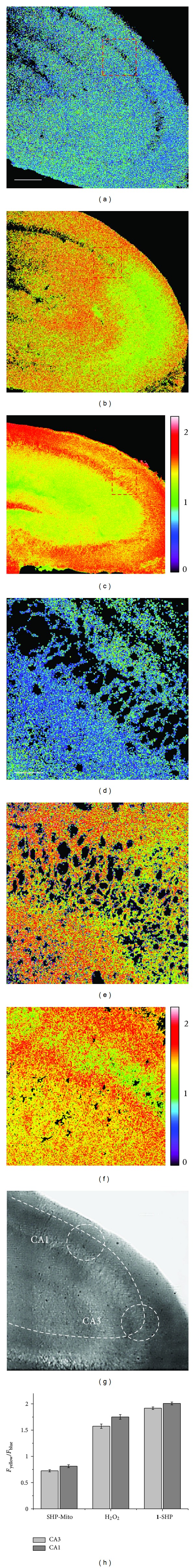
Ratiometric TPM images of a rat hippocampal slice stained with (a) 20 *μ*M SHP-Mito and (c) **1**-SHP and (b) pretreated with 1 mM H_2_O_2_ for 30 min before labeling with 20 *μ*M SHP-Mito. Ten ratiometric TPM images were accumulated along the *z*-direction at the depths of approximately 90–180 *μ*m with magnification at 10x. (d–f) Enlarged images of (a–c) at 120 *μ*m depth with 40x magnification. (g) Approximate positions (dotted circles) used for measurements of emission ratios in the CA3 and CA1. (h) Average *F*_yellow_/*F*_blue_ in (a–c). The TPEF was collected at two channels (blue = 400–470 nm and yellow = 530–600 nm) upon excitation at 750 nm with fs pulse. Scale bars: 300 *μ*m (a) and 75 *μ*m (d) [11].

The utility of SHP-Mito in the tissue imaging was established by a similar protocol as described earlier. TPM image of fresh hippocampal slices from a 2-day-old rat labeled with 20 *μ*M SHP-Mito revealed that H_2_O_2_ is more or less evenly distributed in both the CA1 (*F*_green_/*F*_blue_ = 0.81) and CA3 (*F*_green_/*F*_blue_ = 0.72) regions (Figure 4(a)) at depths of 90–180 *μ*m. Upon treatment of the tissue with increasing amounts of H_2_O_2_, the ratio increased gradually to 1.57 (CA3) and 1.75 (CA1) at 1 mM H_2_O_2_, which lie between those measured in SHP-Mito- and **1**-SHP-labeled tissues (Figure 4(h)). Hence, SHP-Mito is responsive to rises in H_2_O_2_ in tissue. Moreover, the ratiometric image at higher magnification clearly showed the H_2_O_2_ distribution in the individual cells in the CA3 region at a depth of 120 *μ*m (Figures 4(d)–4(f)). Hence, SHP-Mito is capable of detecting mitochondrial H_2_O_2_ in live tissues using TPM [11].

## 4. Two-Photon Probe for Mitochondrial Thiols 

**Scheme 3:**
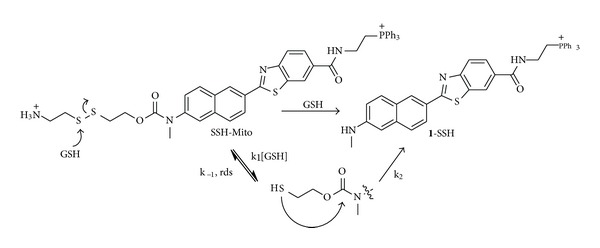
Structures of SSH-Mito and mechanism of the GSH-induced reduction of SSH-Mito [12].

Intracellular thiols such as cysteine (Cys), homocystein (Hcy), and glutathione (GSH) play vital roles in biology [3–5]. They maintain higher-order structures of proteins and control redox homeostasis through the equilibrium between thiols (RSH) and disulfides (RSSR) [46]. In mitochondria, GSH plays a key role in maintaining the redox environment to avoid or repair oxidative damage leading to dysfunction and cell death [47]. Mitochondrial GSH (mGSH) exists predominantly in the reduced form, with the GSH : GSSG ratio being greater than 100 : 1 [48]. To detect GSH deep inside live tissues, we have developed a TP probe (SSH-Mito, Scheme 3) [12].

SSH-Mito is a TP ratiometric probe based on the internal charge transfer (ICT) process [31]. The emission spectra of SSH-Mito showed a gradual increase at 525–575 nm (*F*_yellow_) with a concomitant decrease at 425–475 (*F*_blue_) nm in the presence of 10 mM GSH in MOPS buffer (30 mM, pH 7.4) (Table 1). The reaction followed 2nd-order kinetics with *k*_2_ = 2.3 ×10^−2^  M^−1^ s^−1^ (Scheme 3) [12]. This indicates that the reaction proceeds by the rate-limiting attack of GSH at the disulfide bond followed by the cleavage of the C-N bond to afford **1**-SSH (Scheme 3). SSH-Mito exhibited strong response toward thiols, including GSH, Cys, dithiothreitol (DTT), 2-mercaptoethanol (2-ME), and 2-aminoethanethiol (2-AET), and a negligible response toward amino acids without thiol groups (glu, ser, val, met, ala, and ile), metal ions (Na^+^, K^+^, Ca^2+^, Mg^2+^, and Zn^2+^), and H_2_O_2_ and was pH insensitive at the biologically relevant pH range [19]. Moreover, *F*_yellow_/*F*_blue_, the ratio of the intensities at 425–475 nm (*F*_blue_) and 525–575 nm (*F*_yellow_), increased by 45-fold in the presence of 10 mM GSH (Table 1). Further, the TP action spectra of SSH-Mito and **1**-SSH indicate Φ*δ*_max⁡_ values of 80 and 55 GM at 740 and 750 nm, respectively, which are comparable to those of existing TP probes (Table 1) [15]. These results predict a bright ratiometric TPM image of the living specimens stained with SSH-Mito. 

**Figure 5:**
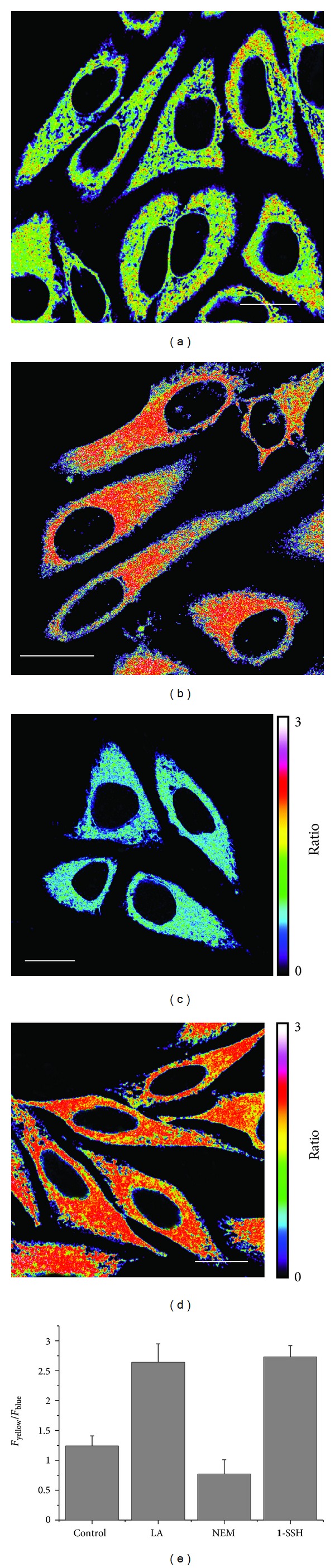
(a–d) Pseudocolored ratiometric TPM images (*F*_yellow_/*F*_blue_) of HeLa cells incubated with 5 *μ*M SSH-Mito (a) and **1**-SSH (d) and pretreated with lipoic acid (500 *μ*M) for 1 day (b) and NEM (100 *μ*M) for 30 min (c) before labeling with SSH-Mito. (e) Average *F*_yellow_/*F*_blue_ intensity ratios in figures (a–d). Images were acquired using 740 nm excitation and fluorescent emission windows: blue = 425–475 nm and yellow = 525–575 nm. Scale bar = 20 *μ*m. Cells shown are representative images from replicate experiments (*n* = 5) [12].

SSH-Mito was found to locate predominantly in mitochondria as revealed by the colocalization experiment with MitoTracker Red (*A* = 0.85) [12]. Upon 740 nm TP excitation, the ratio image of the SSH-Mito-labeled HeLa cells, constructed from two collection windows, gave an average emission ratio of 1.24 (Figures 5(a) and 5(e)). More importantly, SSH-Mito was responsive to the changes in the thiol concentration; the *F*_yellow_/*F*_blue_ ratio increased to 2.64 when the cells were preincubated for 1 day with *α*-lipoic acid (Figure 5(b)), which increases GSH production [49], and the value was nearly identical to those obtained with **1**-SSH (2.73, Figure 5(d)). The *F*_yellow_/*F*_blue_ ratio also decreased to 0.77 upon treatment with *N*-ethylmaleimide (NEM) (Figure 5(c)), a well-known thiol-blocking reagent [50]. Further, SSH-Mito is nontoxic to cells during the imaging experiments as determined by a CCK-8 kit. These results establish that SSH-Mito is capable of detecting mitochondrial thiol in the live cells.

**Figure 6:**
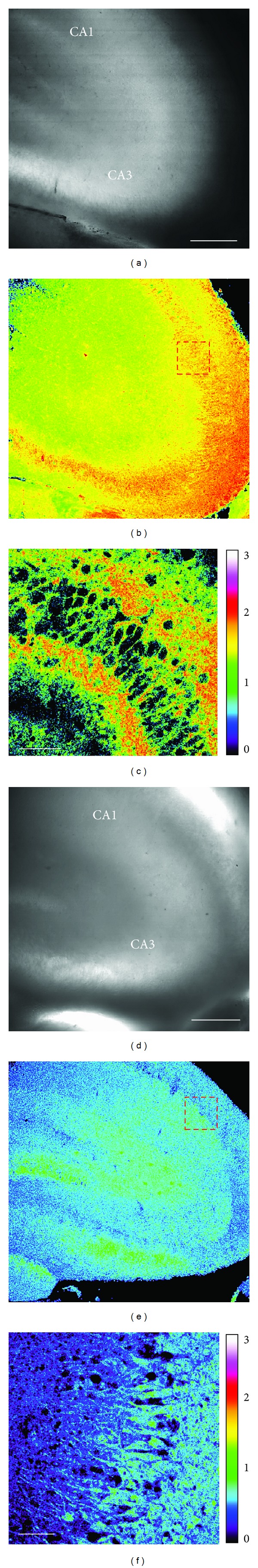
Images of a rat hippocampal slice stained with 20 *μ*M SSH-Mito for 2 h. (a and d) Bright-field images of the CA1-CA3 regions. (b and e) Ratiometric TPM images of a fresh rat hippocampal slice (b) nontreated and (e) pretreated with NEM (100 *μ*M) for 30 min before labeling with SSH-Mito. Ten ratiometric TPM images were accumulated along the *z*-direction at the depths of approximately 90–190 *μ*m with magnification at 10x. (c and f) Enlarged images of red box in (b) and (e) at 120 *μ*m depth with 40x magnification. The TPEF was collected at two channels (blue = 425–475 nm and yellow = 525–575 nm) upon excitation at 740 nm with fs pulse. Scale bars: 300 *μ*m (a and d) and 75 *μ*m (c and f) [12].

The TPM image of fresh hippocampal slices from a 14-day-old rat labeled with 20 *μ*M SSH-Mito revealed that thiols are more or less evenly distributed in both CA1 and CA3 regions at depths of 90–190 *μ*m (Figure 6(b)). Moreover, the image at a higher magnification clearly shows the thiol distribution in the individual cells in the CA1 region with an average emission ratio of 1.66 at a 120 *μ*m depth (Figure 6(c)). Upon treatment of the tissue with 100 *μ*M NEM for 30 min, the ratio decreased to 0.85 (Figure 6(f)). It is worth noting that the changes in the emission ratios measured deep inside the tissue slice are comparable to those in the cells. Hence, SSH-Mito is clearly capable of detecting mitochondrial thiols in live tissues using TPM [12].

## 5. Conclusions 

We have developed a series of mitochondrial-targeted TP probes that can selectively detect the Zn^2+^, H_2_O_2_, and thiols, respectively, in live cells and tissues. All of these probes have been developed by linking mitochondrial targeting site and specific receptors at the opposite ends of the TP fluorophore. They show significant TP action cross-sections, a marked turn-on or ratiometric response upon reaction with the analytes, good mitochondrial localization, low cytotoxicity, insensitivity to pH in the biologically relevant pH range, and can visualize the distribution and changes in mitochondrial Zn^2+^, H_2_O_2_, and thiols levels, respectively, in live tissues at more than 100 *μ*m depth by TPM without interference from other biologically relevant species. 

## Figures and Tables

**Table 1:** Photophysical data for TP probes [10–12, 19].

Probe	*λ*_max⁡_^(1)^/*λ*_max⁡_^fl^ ^[a]^	Φ^[b]^	*K*_d_^OP^/*K*_d_^TP^ ^[c]^	FEF^[d]^	*R*_max⁡_/*R*_min⁡_^[e]^	*λ*_max⁡_^(2)^ ^[f]^	*δ*Φ^[g]^
**SZn-Mito** ^ [h]^	388/500	0.15	3.1/3.1 nM	7 (7)	—	—	—
**SZn-Mito**/Zn^2+[h]^	375/493	0.92				760	75
**SZn2-Mito** ^ [i]^	413/536	0.0048	1.4/1.4 nM	70 (68)	—	—	—
**SZn2-Mito**/Zn^2+[i]^	395/536	0.33				750	155
**SSH-Mito** ^ [j]^	338/462	0.82	—	—	45(40)	740	80
**SSH-Mito/**GSH (**1**-SSH)^[j]^	383/545	0.12				750	55
**SHP-Mito** ^ [k]^	342/470	0.13	—	—	75(40)	740	11
**SHP-Mito/**H_2_O_2 _(**1**-SHP)^[k]^	383/545	0.12				750	55

^[a]^ *λ*_max⁡_ of the one-photon absorption and emission spectra in nm. ^[b]^Fluorescence quantum yield. ^[c]^Dissociation constants measured by one- (*K*_d_^OP^) and two-photon (*K*_d_^TP^) processes, except otherwise noted. ^[d]^Fluorescence enhancement factor, (*F* − *F*_min⁡_)/*F*_min⁡_, measured by one-photon processes. The number in parentheses is measured by two-photon process. ^[e]^ *R*_max⁡_ and *R*_min⁡_ represent the maximum and minimum ratios of *F*_yellow_/*F*_blue_, where *F*_yellow_ and *F*_blue_ are the fluorescence intensities measured at 425–475 nm (*F*_blue_) and 525–575 nm (*F*_yellow_), respectively. The number in parentheses was measured by two-photon process. ^[f]^ *λ*_max⁡_ of the two-photon excitation spectra in nm. ^[g]^The peak two-photon action cross-section in 10^−50^ cm^4^ s/photon (GM). ^[h]^Measured in MOPS buffer (30 mM MOPS, 100 mM KCl, 10 mM EGTA, pH 7.2) in the absence and presence (120 nM) of free Zn^2+^. ^[i]^Measured in HEPES buffer (50 mM HEPES, 100 mM KCl, 10 mM NTA, pH 7.4) in the absence and presence (47 nM) of free Zn^2+^. ^[j]^Measured in MOPS buffer (30 mM MOPS, 100 mM KCl, pH 7.4) before and 2 h after addition of 10 mM GSH. ^[k]^Measured in MOPS buffer (30 mM MOPS, 100 mM KCl, pH 7.4) before and 1 h after addition of 1 mM H_2_O_2_.
